# *Off-label* Use of Gastrointestinal Multiplex PCR Panel for the Diagnosis of Amoebic Liver Abscess: A Three-case Pediatric Report

**DOI:** 10.1093/ofid/ofaf631

**Published:** 2025-10-15

**Authors:** Michael Muñoz-Rosa, Natalia Arango-Serna, Juan Mesa-Monsalve, Alejandro Diaz-Diaz

**Affiliations:** Division of Pediatric Infectious Diseases, Department of Pediatrics, Universidad CES, Medellin, Colombia; Department of Pediatrics, Corporación Universitaria Remington, Medellín, Colombia; Hospital General de Medellín, Clínica las Americas Auna, Medellín, Colombia; Hospital General de Medellín, Hospital Pablo Tobón Uribe, Medellín, Colombia

**Keywords:** diagnosis, *Entamoeba histolytica*, liver abscess, molecular, pediatric

## Abstract

Diagnosis of amoebic liver abscess is challenging. A multiplex polymerase chain reaction panel, traditionally employed for the detection of enteric pathogens in stool, proved useful for the rapid diagnosis in 3 pediatric patients when used off-label on liver abscess fluid. While this approach enabled timely and targeted antiamoebic therapy, highlighting its potential in high-burden, resource-limited settings, it represents an unvalidated use of the technology that requires further investigation.

Liver abscesses, which can be caused by bacteria or parasites, are a major cause of morbidity and mortality in low- and middle-income settings. It is the main extraintestinal complication of *Entamoeba histolytica* infection. When the infection spreads, it can lead to severe complications such as intestinal necrosis, peritonitis, perforation, and toxic megacolon [1].

Amoebic liver abscess occurs in approximately 1% of infected individuals and represents the second leading cause of death from parasitic diseases worldwide [1]. While immunodiagnosis is crucial for identifying the infection (with reported sensitivity and specificity over 95%), there are challenges that limit its effectiveness in regions with a high prevalence of *E. histolytica* [1, 2]. Antibodies typically become detectable after the first week of symptoms, but they may be falsely negative if tested shortly after the onset of disease [1]. These infections can be detected using enzyme immunoassays and indirect hemagglutination tests, but their use is constrained in areas with high infection rates due to limited availability [1, 2].

Patients with intestinal amebiasis are predominantly asymptomatic carriers. In the presence of positive immunological tests, this can lead to false positives and errors in diagnosing amebic liver abscess. Recent developments in molecular diagnostic tools, such as commercial panels for the rapid detection of pathogens in various samples, have resulted in a paradigm shift in microbiology and clinical practice [1, 2].

The FilmArray Gastrointestinal^®^ (GIFA) panel (Biofire Inc., a property of Biomerieux, France) detects over 20 gastrointestinal pathogens in fecal samples, including *E. histolytica* [3]. However, since it detects genetic material, its use in other types of samples is plausible. There is limited but growing evidence of off-label use of the FilmArray platform for samples other than those approved by the manufacturer [4–6]. Here, we report our experience in 3 children with clinical and ultrasound diagnosis of liver abscess, whose amebic etiology was confirmed with GIFA performed directly on the surgical specimen.

## PATIENTS AND METHODS

### Patient #1

A 5-year-old previously healthy preschooler presented with a 12-day history of abdominal pain, a fever of 40°C, and a headache. On admission, leptospirosis was suspected, and treatment with ceftriaxone was initiated. Imaging revealed a single 6.1 cm liver abscess in segment VII. Consequently, the treatment spectrum was broadened by adding metronidazole to the initial empirical therapy. Due to the abscess's size, surgical drainage was performed, yielding 100 cc of purulent fluid. Fecal microscopy studies and bacterial cultures of the abscess were negative. GIFA performed on the surgical fluid detected *E. histolytica*. Based on these findings, ceftriaxone was discontinued while metronidazole therapy was maintained, and the patient subsequently demonstrated satisfactory clinical progress.

### Patient #2

A 5-year-old previously healthy preschooler presented with a 10-day history of abdominal pain, a fever of 39.3°C, and recurrent vomiting. On admission, a urinary tract infection was suspected due to fever, tachycardia, abdominal pain, and symptoms of urinary irritation, and empirical antibiotic treatment with amikacin was initiated. Because of the persistence of abdominal pain, an abdominal ultrasound was performed, revealing an 8.6 cm liver abscess in segment VIII. Consequently, the treatment regimen was broadened to include cefotaxime and metronidazole. Percutaneous drainage was carried out, yielding 20 cc of purulent fluid. Routine studies, including microbiology, pathology, and fecal microscopy, were all negative. Therefore, GIFA was performed on the fluid, which detected *E. histolytica*. Management was adjusted to include metronidazole, and the previous antibiotic treatment was discontinued. The patient made a complete recovery.

### Patient #3

A 17-month-old infant from a rural area without access to clean drinking water presented with a 3-day history of irritability, subjective fever, and dysentery. Sepsis of gastrointestinal origin was suspected based on fever, tachycardia, tachypnea, and distal hypoperfusion, and empirical treatment with piperacillin–tazobactam was initiated. Abdominal ultrasound revealed right basal pneumonia and a 3.6 cm liver abscess in segment VII. Metronidazole was added to the treatment regimen. Initially, medical treatment was administered as the patient did not meet the criteria for abscess drainage. However, the patient's condition deteriorated, with elevated acute phase reactants and an increase in the size of the abscess, prompting the continuation of empirical therapy and the decision to perform percutaneous drainage of the abscess. During this procedure, 60 cc of purulent fluid was obtained. Microbiology and pathology studies were negative. GIFA was performed and tested positive for *E. histolytica*. Consequently, treatment was adjusted by discontinuing piperacillin–tazobactam. The patient showed satisfactory progress after the drainage.

## DISCUSSION

We report 3 pediatric cases of amebic liver abscesses, with the etiology confirmed through off-label use of the GIFA panel directly on liver abscess specimens, rather than on fecal material as recommended by the manufacturer. This novel method facilitated an accurate and rapid diagnosis. It demonstrated the crucial role of the GIFA panel in enabling the implementation of effective targeted therapy, which resulted in favorable clinical outcomes.

The differential diagnosis of liver abscesses is complex due to the wide range of possible bacterial, fungal, and parasitic pathogens [1, 7]. Imaging techniques are highly sensitive for identifying lesions but are less effective for determining the etiology. Suspicion of amebiasis arises from a thorough analysis of the patient's risk factors, travel history to hyperendemic areas, and hygiene practices. This raises clinical suspicion in cases of single liver abscesses located in the right lobe that do not respond to empirical antibiotic treatment [1, 7].

Amoebic liver abscess is a life-threatening complication of invasive *E. histolytica* infection, requiring early diagnosis and treatment in endemic regions such as Latin America. Due to the high prevalence in this area, up to 40% of individuals may have positive immunological tests from past infections. This complicates diagnostic confirmation by these methods, although a negative result has a high negative predictive value (NPV) and is useful for ruling out amebic etiology [1, 7].

These 3 cases highlight the uncertainties in the etiologic diagnosis of liver abscesses. Conventional methods, such as microscopy and serology, are not entirely reliable. Additionally, immunodiagnostic tests are often not routinely available in many hospitals, and waiting times for results can be long, which is not ideal for clinical decision-making [1]. In our patients, careful history-taking revealed non-potable water consumption, but stool microscopy was negative for parasites. This illustrates the diagnostic challenges and missed opportunities in patient care [1]. The clinical characteristics and treatment details of the 3 patients are summarized in Table 1.

**Table 1.** Clinical Characteristics and Treatment Details of 3 Pediatric Patients With Amoebic Liver Abscess, Confirmed Using Off-Label Filmarray Gastrointestinal^®^ (Gifa) Panel on Liver Abscess Fluid

	Patient #1	Patient #2	Patient #3
Sex	Male	Female	Male
Age	5 years	5 years	17 months
History	Occasional non-potable water consumption.	Occasional non-potable water consumption.	Occasional non-potable water consumption.
Symptoms	Fever (40°C), abdominal pain, headache.	Fever (40°C), abdominal pain, vomiting.	Subjective fever, vomiting, high output diarrhea.
Symptom days	12 days of symptoms.	10 days of symptoms.	3 days of symptoms.
Initial empirical treatment	Ceftriaxone + Metronidazole.	Cefotaxime + Metronidazole.	Piperacillin/Tazobactam + Metronidazole.
Targeted treatment	Metronidazole (7 days)	Metronidazole (9 days)	Metronidazole (15 days)
Time to abscess drainage	4 days	7 days	18 days

PCR-based tests for detecting DNA (deoxyribonucleic acid) are considered the reference standard for diagnosing amebiasis [8], especially when microscopy results are negative, as they require experienced personnel [1, 9, 10]. A major challenge is the lack of specific commercial tests and their standardization for detecting *E. histolytica*, since few patients with this pathogen were included in the validation [8, 9]. In contrast, multiplex PCR molecular panels that detect the most relevant pathogens in gastrointestinal infections, including *E. histolytica*, are only approved for use with fecal samples and not other fluids or tissues [3, 10, 11].

In 2007, N. Ahmad et al. demonstrated that PCR detection of *E. histolytica* in liver abscess specimens is superior to amebic antigen detection (sensitivity 97% vs. 40%). This finding is consistent with Othman et al. [11], where specific PCR was also superior to serum antibody detection by indirect hemagglutination [12]. Other reports in the literature discuss unconventional uses of multiplex gastrointestinal PCR in liver abscess fluid, which led to rapid and accurate diagnoses, helping overcome the diagnostic barriers associated with this disease [12, 13]. The use of the gastrointestinal (GI) panel has been documented not only in non-approved samples, but there are also data on the use of sepsis, pneumonia, and meningitis/encephalitis panels in sample types other than those indicated by the manufacturer.

For example, Michos et al. [4] highlighted the usefulness of the sepsis panel in synovial and pleural fluids for the timely detection of *S. pyogenes* and *S. pneumoniae*, respectively, allowing rapid diagnosis from direct clinical specimens and benefiting patient care. Additionally, Londoño-Ruiz et al. [6] presented a series of 17 patients where the pneumonia panel from Biofire Inc., used directly on pleural fluid, was useful for etiological detection in 94% of cases and modified clinical management in 52%, demonstrating its application in optimizing patient care. There are also reports of off-label use of the meningitis/encephalitis panel, as shown by Garcia-Clemente et al., who detected *Neisseria meningitidis* in skin biopsy and whole blood samples from a patient with acute meningoencephalitis [5].

In this context, DNA detection techniques are novel, especially when microscopy and immunological tests are negative. Additionally, multiple gastrointestinal panels could detect other pathogens in liver abscesses, aiding in differential diagnosis [3, 9].

Despite the successful outcomes in these 3 cases, it is imperative to emphasize a significant limitation: the use of the GIFA panel directly on liver abscess fluid is an off-label application. Since the test is not validated for this type of specimen, its true diagnostic performance—including sensitivity, specificity, and predictive values—remains unknown. Therefore, while our experience suggests a potential solution for rapid diagnosis, these results must be interpreted with caution. Future prospective studies are essential to critically evaluate and validate the non-endorsed use of multiplex gastrointestinal panels in such specimens. Until such data become available, these molecular methods, although promising, should be considered complementary tools, particularly when conventional diagnostics are inconclusive.

It should be noted that these multiplex panels could be the only option for rapid and reliable diagnosis of this neglected disease, overcoming barriers faced by vulnerable populations in our countries, as demonstrated by our experience. Molecular methods, renowned for their high sensitivity, can effectively address diagnostic challenges when applied in the appropriate clinical context, particularly when conventional methods prove insufficient.
